# Use of pooled serum samples to assess herd disease status using commercially available ELISAs

**DOI:** 10.1007/s11250-021-02939-1

**Published:** 2021-10-09

**Authors:** Juan Heberth Hernandez-Medrano, Luis Fernando Espinosa-Castillo, Ana D. Rodriguez, Carlos G. Gutierrez, Wendela Wapenaar

**Affiliations:** 1 Department of Reproduction, Faculty of Veterinary Medicine and Zootechnics, National Autonomous University of Mexico, Ciudad Universitaria, 04510 Mexico City, Mexico; 2 Present Address: Division of Child Health, Obstetrics and Gynaecology, School of Medicine, D Floor East Block, Queen’s Medical Centre, University of Nottingham, Derby Road, Nottingham, NG7 2UH UK; 3 School of Veterinary Medicine and Science, University of Nottingham, Loughborough, LE12 6LJ UK

**Keywords:** Pooled serum, ELISA, Bovine viral diarrhoea, BVD, Infectious bovine rhinotracheitis, IBR, *Neospora caninum*, Enzootic bovine leukosis, EBL

## Abstract

Pooled samples are used in veterinary and human medicine as a cost-effective approach to monitor disease prevalence. Nonetheless, there is limited information on the effect of pooling on test performance, and research is required to determine the appropriate number of samples which can be pooled. Therefore, this study aimed to evaluate the use of pooled serum samples as a herd-level surveillance tool for infectious production-limiting diseases: bovine viral diarrhoea (BVD), infectious bovine rhinotracheitis (IBR), enzootic bovine leukosis (EBL) and *Neospora caninum* (NC), by investigating the maximum number of samples one can pool to identify one positive animal, using commercial antibody-detection ELISAs. Four positive field standards (PFS), one for each disease, were prepared by pooling highly positive herd-level samples diagnosed using commercially available ELISA tests. These PFS were used to simulate 18 pooled samples ranging from undiluted PFS to a dilution representing 1 positive in 1,000 animals using phosphate-buffered saline as diluent. A 1:10 dilution of the PFS resulted in positive results for IBR, BVD and EBL. Moreover, for IBR and BVD, results were still positive at 1:100 and 1:30 dilutions, respectively. However, for NC, a lower dilution (8:10) was required for a seropositive result. This study indicates that, at herd-level, the use of pooled serum is a useful strategy for monitoring infectious diseases (BVD, IBR and EBL) but not NC, using readily available diagnostic assays.

## Introduction

Sample pooling is a method used in human and veterinary medicine to obtain disease information in a cost-effective way, where individual results are not required and the diagnostic method is particularly labour intensive. Pooled samples are commonly used for surveillance of infectious diseases, to monitor specific pathogens and to assist in population-based management decisions (Department for the Environment Food and Rural Affairs 2015; Lindberg and Alenius 1999; Niskanen et al. 1991; Nylin et al. 2000; Sayers et al. 2015).

The use of sample pooling has been reported for molecular and immune-based diagnostic methods. For example, PCR tests were used to estimate the presence of bovine viral diarrhoea virus (BVD) in serum pools of 30 animals at auction markets (Smith et al. 2008) and pooled calf ear notch samples were used as a rapid method to detect BVD-positive animals (Kennedy et al. 2006). Pooled faeces and bulk milk samples have been used to detect the presence of *Salmonella* spp. in calves (Singer et al. 2006) and *Staphylococcus aureus* in cows with mastitis (Ronco et al. 2018). An Australian study evaluated the use of pooled serum for the identification of BVD and demonstrated that a single high antibody-positive individual could give a positive result in pools of up to 128 animals, while a single weak-positive animal would generate a positive result in pools of up to eight animals (Lanyon et al. 2014).

A similar approach in human medicine has been reported using serum sample *pooling* for the detection of HIV-positive blood used for transfusion (Soroka et al. 2003). Moreover, a Danish study evaluated the use of serum sample pooling using three diagnostic methods (ELISA, line blot, immunofluorescence microscopy) and four diseases (Sjögren’s syndrome, systemic sclerosis, systemic lupus erythematosus or rheumatoid arthritis) and confirmed that this approach can be used as a quick and efficient screening method (Sternbæk et al. 2017).

Despite the effectiveness of a PCR in the detection of pathogens when samples are pooled, this method requires more specialised equipment and training, compared to the enzyme-linked immunosorbent assay (ELISA). ELISAs are relatively easy to perform, cost-effective, readily available and validated for a wide range of diseases using samples such as milk, plasma and serum. However, when a large number of samples are assayed, the cost of analysing individual samples can be prohibitive; therefore, sample pooling can be a more appropriate approach. For example, an ELISA to detect IBR-glycoprotein E was able to differentiate between naturally infected and vaccinated cattle using pooled serum and milk samples, with a sensitivity (Se) of 100% (Muratore et al. 2017).

The chance of a decreased test Se is a potential disadvantage of pooling samples and may lead to an increase in false-negative results due to the dilution of antibodies or antigens when only a few animals are infected. In contrast, specificity (Sp) of an ELISA, associated with false-positive results due to, for example, cross-reaction with other antigens, is less likely to be affected in pooled compared to individual samples.

BVD, IBR, EBL and NC are production limiting diseases which affect the beef and dairy industry worldwide. This study aimed to evaluate the influence of dilution when pooling bovine serum samples on the ability to assess the BVD, IBR, enzootic bovine leukosis (EBL) and *Neospora caninum* (NC) herd prevalence using commercially available antibody-detection ELISAs. In addition, the study aimed to provide evidence for a recommended maximum number of animals to contribute to a pooled sample and provide a conservative estimate of the apparent between herd prevalence of these four production-limiting diseases in the major farming provinces in Mexico, taking into account the reduced sensitivity when using pooled samples. 

## Material and methods

The study was reported following the Standards for Reporting Diagnostic accuracy studies (STARD) guidelines (Bossuyt et al. 2015). The study was approved by the Internal Committee for the Care and Use of Animals (CICUA) of the Faculty of Veterinary Medicine and Zootechnics of the National Autonomous University of Mexico (FMVZ-UNAM).

### Sample collection

Samples were obtained from the National Bovine Serum Bank (NBSB) stored at the Department of Reproduction of the FMVZ-UNAM. For this study, 5,482 individual serum samples from a total of 514 herds were selected from five states in Mexico’s tropical region which hold more than 50% of the national herd: Chiapas (111 herds), Guerrero (94 herds), Tabasco (130 herds), Tamaulipas (89 herds) and Veracruz (90 herds). All cattle were grazing beef or dual-purpose breeding-age females with no history of previous vaccinations.

Herd sampling was carried out using a stratified randomised sampling method with state and herd size as strata based on the Neyman proportional allocation method (Bankier 1988). Following this methodology, a total of 301,799 cows in 6,529 farms distributed across Mexico were sampled. Serum samples were taken as part of a national survey of the reproductive status of grazing herds. Sampling methodology was based on the National Herd Inventory 2009 published by the Mexican Ministry of Agriculture for farms with more than 20 head (hd). Herds were classified according to size into small (20–35hd), medium (36–100hd) and large (> 101hd). The producers were randomly selected to participate as part of a National Reproductive Survey supported by the Ministry of Agriculture and FMVZ-UNAM. Producers’ participation was not mandatory but high as they received useful information about fertility and health status of the herd in return for their participation.

All animals were sampled by trained veterinarians between 2009 and 2012.

For each herd, a convenience sample of 10 to 12 female animals of breeding age was blood sampled (10 ml) by puncture of coccygeal vessels. Blood samples were centrifuged on site at 3500 rpm for 10 min, serum harvested and transported to the NBSB under refrigeration (4–8 °C). In some cases (~ 15% of total), whole blood samples were posted under refrigeration at 4–8 °C. The time between sampling and processing of these samples was 2–3 days and samples were centrifuged on arrival. All serum samples were kept at − 20 °C until analysis between 2016 and 2017.

Serum samples were thawed once to prepare one pooled sample per herd mixing 100 µl of each individual sample, obtaining a total of 514 herd samples. When sufficient serum was available from each individual sample, 10 samples were chosen at random to establish one pooled herd sample. When 12 individual samples were available, and the quantity of serum was low, all 12 individual samples were used.

The number of pooled herd samples analysed differed per disease; 506, 510, 514 and 469 pooled herd samples were analysed for BVD, IBR, EBL and NC, respectively.

### Diagnostic methods

Samples were analysed using four commercially available ELISAs: BVDV Total Ab Test, IBR gB X3 Ab Test, Leukosis Blocking Ab Test (IDEXX Laboratories, Westbrook, Maine, USA) and CIVTEST® BOVIS NEOSPORA (HIPRA Laboratories, Amer, Spain). Analysis was performed by trained laboratory personnel, with validity of all ELISAs evaluated following manufacturers’ recommendations (Table 1). All samples were analysed in duplicate.

**Table 1:** Test characteristics of bovine viral diarrhoea (BVD), infectious bovine rhinotracheitis (IBR), enzootic bovine leukosis (EBL) and *Neospora caninum* (NC) ELISAs and result interpretation according to manufacturers’ recommendations

Disease	ELISA type	Outcome measure	Calculation*	Se (%)*	Sp (%)*	Cut-off points*		
						Positive	Negative	Inconclusive
BVD	Indirect	Sample to positive ratio (S/P)	S/P = (OD − ODneg)/(ODpos − ODneg)	96.3	100	> 0.2	≤ 0.2	N/A
IBR	Blocking	% Blocking	% Blocking = [(ODneg-OD)/(ODneg)]*100	97.4	100	> 55%	< 45%	45–55%
EBL	Competitive blocking	% Blocking	% Blocking = (OD/ODneg)*100	100	99.8	< 40%	≥ 40%	N/A
NC	Indirect	Relative index percent (RI%)	RI% = [(OD − ODneg)/(ODpos − ODneg)]*100	95.7	100	> 10%	< 6%	6–10%

Key: *BVD*, bovine viral diarrhoea; *IBR*, infectious bovine rhinotracheitis; *EBL*, enzootic bovine leukosis; *NC*, *Neospora caninum*; *Se*, sensitivity; *Sp*, specificity; *OD*, optical density of sample; *ODneg*, OD of negative control; *ODpos*, OD of positive control

^*^Values reported by the manufacturer for samples at the individual level

### Positive field standard (PFS)

For each disease, 10 highly positive herd-pooled samples per each of the five Mexican states were selected and pooled into a single positive field standard (PFS). This resulted in four PFS samples, one for each disease (BVD-PFS, IBR-PFS, EBL-PFS and NC-PFS), containing 50 herd-pooled samples each, i.e. 10 herd-pooled samples × 5 states = 50 herd-pooled samples per PFS. This pooling was carried out as part of a separate study which required the availability of large volumes of sample. These four PFS were used in this study to simulate a positive control and were used in the dilution experiment.

### Dilution experiment

For the dilution experiment, the PFS was diluted with PBS (phosphate-buffered saline) to simulate decreasing numbers of seropositive animals in the pooled sample. For BVD and IBR, a total of 18 dilutions ranging from undiluted to 1 in 1000 were prepared; while for EBL and NC, 10 dilutions ranging from undiluted to 1 in 1000 were considered (Table 2). All samples were analysed in duplicate.

**Table 2:** Positive field standard (PFS) dilution steps; PFS was diluted to simulate decreasing numbers of seropositive animals in the pooled samples which were then analysed using a bovine viral diarrhoea (BVD), infectious bovine rhinotracheitis (IBR), enzootic bovine leukosis (EBL) and *Neospora caninum* (NC) commercial ELISAs

	Simulated proportion of positive animals to the total		Dilution nomenclature*	Disease evaluated			
	( +) ve animals	Total		BVD	IBR	EBL	NC
1	10	10	10:10	X	X	X	X
2	9	10	9:10	X	X	X	X
3	8	10	8:10	X	X	X	X
4	7	10	7:10	X	X	X	X
5	6	10	6:10	X	X	X	X
6	5	10	5/:10	X	X	X	X
7	4	10	4:10	X	X	X	X
8	3	10	3:10	X	X	X	X
9	2	10	2:10	X	X	X	X
10	1	10	1:10	X	X	X	X
11	1	20	1:20	X	X		
12	1	30	1:30	X	X		
13	1	40	1:40	X	X		
14	1	50	1:50	X	X		
15	1	70	1:70	X	X		
16	1	80	1:80	X	X		
17	1	100	1:100	X	X	X	X
18	1	1000	1:1000	X	X	X	X

^*^The nomenclature shows the proportion of positive animals (numerator) to the total number of animals (denominator). *X*, dilution tested

### Data analysis

Data analysis was performed using Microsoft Excel 2010 (Microsoft, Redmond, USA) and GraphPad Prism v8.0.0 (GraphPad Software, San Diego, USA). Descriptive statistics, using box-and-whisker plots, were used to describe ELISA results for pooled field samples (*n* = 514). The PFS ELISA results were compared to the corresponding pooled herd field sample range for each disease to evaluate whether they were true representations of a ‘high positive’ result using descriptive statistics by plotting the data and assessing if the PFS ELISA result was within in the range of results of the 514 herd field samples. The ELISA result from each pooled herd sample was used to estimate the herd-level prevalence using 506, 510, 514 and 469 pooled herd samples for BVD, IBR, EBL and NC respectively.

## Results

**Fig. 1:**
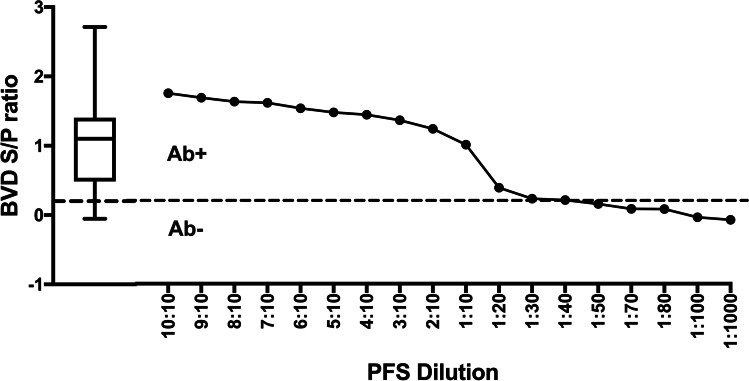
Bovine viral diarrhoea ELISA results (S/P) for pooled field standard (PFS) at different dilutions. The dilution corresponds to the number of positive relative to total number of animals (Table 2). Dotted line indicates recommended cut-off value (S/P ratio = 0.2), separating positive (Ab + ; > 0.2) and negative (Ab − ; < 0.2) reading areas. The box-and-whisker plot shows results from 503 pooled herd samples

**Fig. 2:**
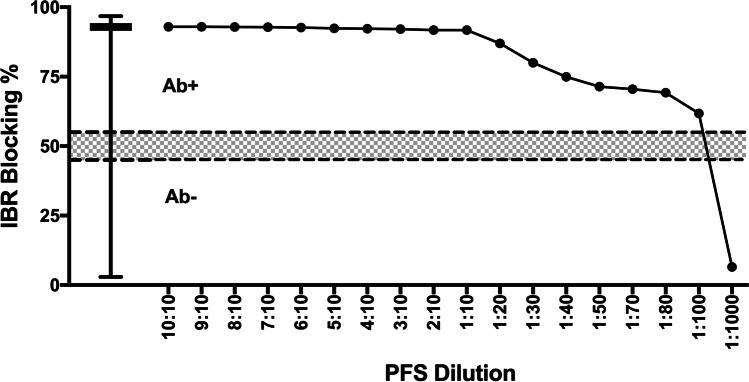
Infectious bovine rhinotracheitis ELISA results (Blocking %) for pooled field standard (PFS) at different dilutions. The ‘PFS Dilution’ corresponds to the number of positive animals relative to total number of animals (Table 2). Dotted lines indicate recommended cut-off values for positive (Ab + ; > 55%) and negative (Ab − ; < 45%) and the shaded area indicates inconclusive readings (IC; > 45% < 55%). The box-and-whisker plot shows results from 506 pooled herd samples

**Fig. 3:**
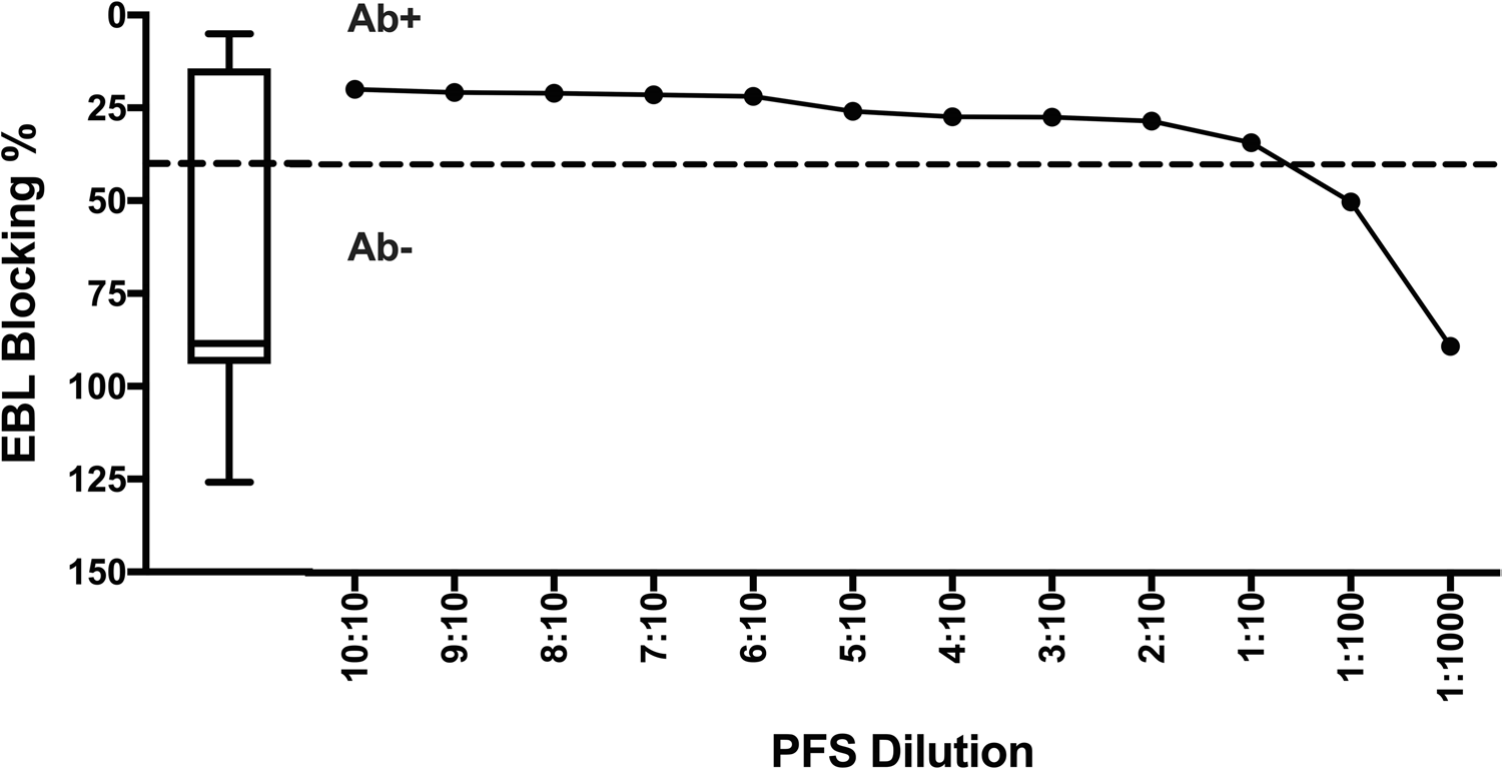
Enzootic bovine leukosis ELISA results (Blocking %) for pooled field standard (PFS) at different dilutions. This is a competitive ELISA with lower values indicating positive results; therefore, the *Y*-axis in the graph is in inverse order. The ‘PFS Dilution’ corresponds to the number of positive relative to total number of animals (Table 2). Dotted line indicates cut-off values (Blocking % < 40), separating positive (Ab +) and negative (Ab −) reading areas. The box-and-whisker plot shows results from 145 pooled herd samples

**Fig. 4:**
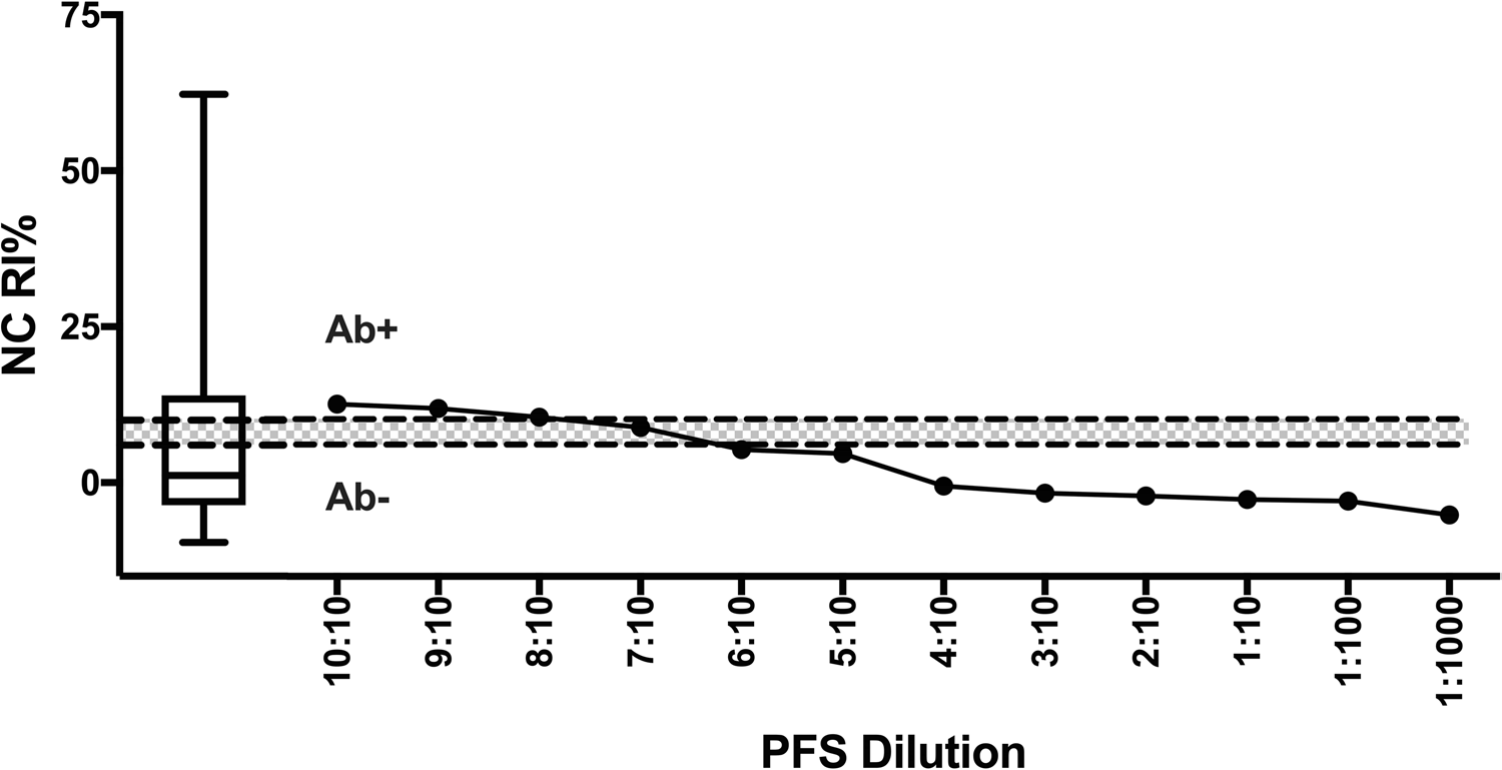
*Neospora caninum* ELISA results (RI%) for pooled field standard (PFS) at different dilutions. ‘PFS Dilution’ corresponds to the number of positive animals relative to total number of animals (Table 2). Dotted lines indicate recommended cut-off values for positive (Ab + ; RI% > 10%) and negative (Ab − ; RI% < 6%) and the shaded area indicates inconclusive readings (IC; > 6% < 10%). The box-and-whisker plot shows results from 152 pooled herd samples tested

Following quality assessment based on manufacturer guidelines, all ELISAs used were considered valid. Undiluted PFS (10:10) showed high positive results in all four ELISAs, confirming they retained their high positivity following pooling (Figs. 1, 2, 3 and 4). With the exception of NC, ELISAs for the three viral pathogens (BVD, IBR, EBL) were able to detect a PFS-pooled sample up to a dilution of 1:10.

The BVD ELISA showed sero-positive results for PFS diluted 1:20 (S/P = 0.38), with a detection limit at a dilution of 1:30 (S/P = 0.22) when the S/P was just outside the recommended cut-off value (S/P < 0.2; Fig. 1).

The IBR ELISA showed the highest detection capacity, being able to detect a positive result at 1:100 dilution (Blocking % = 61%), and the 1:1000 dilution showed a negative result (Blocking % = 6.5%; Fig. 2).

For the EBL ELISA, a positive result was detected at a dilution of 1:10 (Blocking % = 34.1%), which was close to the recommended cut-off value of 40% showing negative results at higher dilutions (Fig. 3).

In the case of NC, the ELISA was able to detect a positive sample when PFS was diluted 8:10, representing 8 positive animals out of 10 (relative index percent, RI% = 10.55%), showing inconclusive or negative results when fewer than eight positive animals were in the pooled sample (Fig. 4). Contrary to the other ELISAs, undiluted PFS-NC showed a lower overall seropositive value (RI% = 12.65%), which was close to the cut-off recommended by the manufacturer (RI% > 10% Fig. 4).

The estimated herd-level seroprevalence for farms included in this study (Table 3) was 80% for BVD (*n* = 405/506), 93.5% for IBR (*n* = 477/510), 36% for EBL (*n* = 185/514) and 34% for NC (*n* = 160/469).

**Table 3:** Estimated herd-level seroprevalence for bovine viral diarrhoea (BVD), infectious bovine rhinotracheitis (IBR), enzootic bovine leukosis (EBL) and *Neospora caninum* (NC) from five states in Mexico’s tropical region: Chiapas (1), Guerrero (2), Tabasco (3); Tamaulipas (4) and Veracruz (5)

Disease	State	Herds tested (*n*)	Positive herds (*n*)	Prevalence (%)	95% CI
BVD	1	105	87	82.9	(76–90)
	2	94	87	92.6	(87–98)
	3	128	86	67.2	(59–75)
	4	89	67	75.3	(66–84)
	5	90	78	86.7	(79–94)
	*Total*	*506*	*405*	*80.0*	*(77–84)*
IBR	1	109	96	88.1	(82–94)
	2	94	92	97.9	(94–101)
	3	128	123	96.1	(92–99)
	4	89	81	91.0	(85–97)
	5	90	85	94.4	(89–99)
	*Total*	*510*	*477*	*93.5*	*(91–96)*
EBL	1	111	78	70.3	(62–79)
	2	94	41	43.6	(33–54)
	3	130	28	21.5	(14–29)
	4	89	23	25.8	(17–35)
	5	90	15	16.7	(9–24)
	*Total*	*514*	*185*	*36.0*	*(32–40)*
*NC*	1	111	41	36.9	(28–46)
	2	92	49	53.3	(43–63)
	3	130	3	2.3	(0–5)
	4	46	22	47.8	(33–62)
	5	90	45	50.0	(40–60)
	*Total*	*469*	*160*	*34.1*	*(29–38)*

There was limited variation in herd prevalence estimates between states for BVD (75 to 93%) and IBR (88 to 99%); more variability was observed for EBL (17 to 70%) and NC (2 to 53%; Table 3).

The ELISA results for all farms tested in the study are presented in the box-and-whisker plots included in Figs. 1, 2, 3 and 4. The ELISA results for the undiluted PFS were higher than the top quartile of herd-pooled samples, confirming a high positive pooled sample (Figs. 1, 2, 3 and 4).

## Discussion

This study supports the use of pooled samples as a diagnostic tool to monitor disease at herd level. For IBR, BVD and EBL, positive results were obtained in samples diluted up to 1 in 10, and even higher for IBR (1 in 100, Fig. 2). However, it was evident that care should be taken when pooling samples to detect seropositive farms to non-viral pathogens such as NC, where more than eight in ten positive animals were needed for a positive ELISA result.

This loss in sensitivity compared to the other assays could be due to different factors such as the type of antigen used, or the characteristics of protozoa in general, with different parasite stages in their lifecycle. A study by Stenlund et al. (1999) confirmed how NC antibody levels change over time during pregnancy in cattle naturally infected with NC and demonstrated a consistent pattern of elevated antibody titres 3 to 5 months before parturition. This variation of antibody levels within an individual animal will influence the overall antibody level in a pooled sample, reducing the ability of an ELISA to identify disease positive animals, which could explain the lack of sensitivity for the pooled NC results.

Two studies comparing ten commercially available NC ELISAs used on individual serum samples (Alvarez-García et al. 2013; Wapenaar et al. 2007) confirmed a high Se and Sp for the NC ELISA used in this study, which corroborates the manufacturer’s reported test characteristics (Table 1). The scarcity of high RI% levels in the 160 Mexican herds (box-and-whisker plot, Fig. 4) matched the poor Se obtained when pooling the PFS for NC. A study carried out by Schares et al. (2004) demonstrated that individual milk samples performed equally to serum samples and are therefore more likely to be beneficial compared to pooling serum, when aiming to make serological surveys for NC more cost-efficient.

IBR antibodies can be detected approximately a week after infection and animals remain infected for life, with reactivation when animals undergo stressful events (Muylkens et al. 2007). The variation of antibody responses and the lack of correlation between antibody presence or absence and the recovery of virus make interpretation based on the presence of antibodies challenging (Huck et al. 1973). Cattle may present a steady increase in the titres of antibodies without showing any clinical signs and, conversely, IBR virus can be re-excreted without increasing antibody titres (Pastoret et al. 1982). Although no vaccine was used in the study herds, the influence of using IBR-marker vaccine on test performance needs consideration. A recent study using BoHV-1 ELISA kits to compare serological conversion between gE and gB antigens reported that gE ELISAs presented a relatively high analytical sensitivity and a good correlation between serum and milk (Tignon et al. 2017). Therefore, a positive result in a pooled sample would support the presence of the virus in the herd, even in the absence of clinical cases.

The IBR ELISA was able to detect a positive result in a 1:100 dilution. This finding is supported by other studies using BTM samples; in the European Union, a maximum BTM pool size of licensed kits corresponds to 50 cows (European Commission (EC) No. 2004/558/EC 2004). The herd-level Se and Sp of an IBR ELISA applied to BTM pools were 55–82% and 97.2–100%, respectively (Elliot 1997; Nylin et al. 2000; Raaperi et al. 2010), with Se shown to increase by repeated testing (Elliot 1997). A low Se was reported in a different study (Schroeder et al. 2012) but improved from 5.4 to 75.7% after a BTM concentration procedure. A new methodology was evaluated by Casarin et al. (2016) changing the enzymatic chemiluminescent substrate HRP conjugate for avidin-nucleic-acid-nanoassembly (ANANAS) which improved the Se fourfold. Although not in commercial use yet, these developments may benefit the use of pooled serum in the future. These developments indicate ongoing improvements of assays to be able to further refine interpretation of ELISA results; results from our study indicate that the ELISA used in this study is suitable to detect IBR antibodies in a herd, using pooled samples of up to 100 animals. This indicates the herd has been exposed to IBR and is likely, due to the epidemiology of the disease to contain latent infected animals.

In pools of 20 animals, it was possible to detect a BVD-seropositive animal, while a pool of 1 in 30 was inconclusive. Sero-conversion is detectable about 3 weeks post-infection, reaching a plateau after 10–12 weeks and animals remain sero-positive long-term. Persistently infected (PI) animals usually do not sero-convert (Sandvik 2005). Although PI animals are sero-negative, and themselves do not interfere with the Se of the ELISA when using serum pools (Graham et al. 2019), care should be taken when interpreting results as a sero-negative pool does not necessarily indicate a BVD-free herd (Drew et al. 1999; Sandvik 2005). When mingled in the herd, the PI will be shedding virus and infect other animals in the herd who, in approximately 3 weeks, can develop antibodies against BVD which contribute to the pooled seropositivity.

Pooling more than 20 samples with the commercial ELISA used in this study can lead to false-negative results, as shown in a previous study where a decrease in Se from 100% (up to 1:8 pools) to ~ 88% in 1:16 and 1:32 pools was observed (Lanyon et al. 2014). Pools of 20 serum samples were successfully used for surveillance in beef herds in Norway, as part of an eradication programme, which resulted in a continuous decrease in BVD-positive herds between 1994 and 2006 (Kampen et al. 2006). Similarly, combination of antibody detection ELISAs with antigen-detection ELISAs and PCR-based tests has proven to be an adequate approach to monitor and control BVD-herd prevalence (Mars and Van Maanen 2005). Therefore, based on our results and those reported by others, pooled serum samples can be a reliable and economical surveillance tool on their own or in combination with other methodologies.

Enzootic bovine leukosis is usually a life-long infection, with a persistent immune response observable 3 weeks post-infection (OIE 2012), with most animals showing a subclinical presentation (Bartlett et al. 2014). In our study, it was possible to detect EBL in a 1:10 dilution, similar as reported by Kuczewski et al. (2018). However, a study using BTM detected positive results at higher dilutions, between 1:50 and 1:200, using two ELISAs for the detection of antibody against glycoprotein gp51 (Ridge and Galvin 2005). Similar results would be expected for pooled serum samples, as antibody titres against EBL are comparable between milk and serum (Evermann et al. 2019). The ELISA used in this study detected the same glycoprotein gp51, and further work would be needed to explain the less sensitive results observed. The main source of EBL transmission is iatrogenic (Benitez et al. 2019), and despite its low mortality (Bartlett et al. 2014), it is worth using pooled samples to monitor the presence of EBL, control transmission and establish adequate strategies for eradication.

Sample pooling has been proposed as a viable and economic method for diagnosing or monitoring disease at a population level in humans (Sternbæk et al. 2017) and animals (Wapenaar et al. 2007). The positive and negative predictive value of such a test using pooled sera relies on the Se and Sp of the test and the prevalence of the disease; if the prevalence is low, the test may not be able to detect a positive pool when there are insufficient positive animals contributing to the pool (Graham et al. 2019; Lanyon et al. 2014). The PFS used in this study represented a ‘high positive’ sample, collected from herds consisting of animals with a high antibody titre and/or a high within-herd prevalence (box-and-whisker plots, Figs. 1, 2, 3 and 4). If these assays are used in the field with herds and animals with potentially lower antibody levels, the ability to detect positive herds may be reduced. Further work using a PFS selected from herds with moderately positive antibody levels could help quantify this impact.

In addition, it is important to consider that in this study only one measurement was carried out for each dilution; although the results appear promising, the robustness of the data would be strengthened by using the same dilution several times. In addition, the study was limited by using PBS as a diluent instead of negative sera; this was chosen to prevent false-positive results *and, in this way,* provide the best estimate of how many animals one could pool to still detect antibodies of one positive animal and positively identify a herd, when using these commercially available ELISAs. Using PBS has likely artificially increased the Sp, and as such decreased false-positive results. An increase in false-positive results may occur when using presumably negative field sera, which may have been false negative themselves or where cross-reaction of antibodies could influence test results.

Further work could explore this limitation, although when using these ELISAs in the field, the main concern is a lack of Se when testing pooled samples, and the Sp was therefore not in focus for this study. Compared to individual animal-level analyses, pooled samples reduce the cost of diagnosis and can therefore help estimate the seroprevalence of diseases in under resourced areas. The use of BTM is often suggested as a useful pooled sample to monitor disease prevalence on a dairy farm; however, it is important to be aware that a BTM excludes non-lactating dairy and beef cattle. Moreover, dairy cows that are diseased or treated with substances that warrant milk withdrawal will not be included in the BTM. Thus, even if the BTM test is analytically sensitive, a herd could be falsely considered as non-infected if infected cows are not included in the pooled sample. Using repeated sampling or pooled serum circumvents this problem. This study provides support that pooled serum samples for IBR, BVD and EBL are a good alternative in beef herds or in areas where access to repeated BTM samples is challenging.

This is the first study to report IBR, BVD, EBL and NC prevalence at herd level in Mexico using pooled serum samples. All four production-limiting diseases showed a high estimated apparent prevalence at herd level: 93.5%, 80%, 36% and 34% for IBR, BVD, EBL and NC respectively. These prevalence estimates were higher for IBR compared to a recent study in dairy herds in Mexico using individual serum samples, who found an overall prevalence of 73%, whereas BVD and NC resulted in a comparable prevalence (79% and 37%, respectively) (Milián-Suazo et al. 2016). Considering the reduced sensitivity when using pooled samples and the various methods used to analyse and report prevalence data, it is challenging to compare data between studies. The true prevalence in Mexican dairy herds may likely be even higher than what is reported here, as due to suboptimal sensitivity false-negative results will outweigh the number of false-positive results in this analysis. However, even considering those uncertainties, both studies report a high prevalence for these three production-limiting diseases, which indicates the importance of veterinary surgeons in Mexico to engage in herd health monitoring and disease control in these regions, to reduce disease transmission and improve cattle health and productivity.

## Conclusion

This study demonstrated disease-specific variability in test performance when using a simulation of pooled serum samples. Estimating herd-level prevalence using pooled serum samples of up to 10 animals for IBR, BVD and EBL appears feasible when one highly positive animal is present in the sample. For NC, pooled serum performed poorly and should not be used in serum pools of more than two animals; individual samples or alternative sources, such as milk samples, may be more appropriate for this pathogen.

## Data Availability

Data is available from the authors upon request.

## Code availability

N/A.
